# Efficacy and safety of stereotactic radiosurgery for pulmonary metastases from osteosarcoma: Experience in 73 patients

**DOI:** 10.1038/s41598-017-14521-7

**Published:** 2017-12-12

**Authors:** Wenxi Yu, Zimei Liu, Lina Tang, Feng Lin, Yang Yao, Zan Shen

**Affiliations:** Department of Oncology, Affiliated Sixth People’s Hospital, Shanghai Jiaotong University, Shanghai, China

## Abstract

Osteosarcoma pulmonary metastases are typically treated with resection and/or chemotherapy. We hypothesize that stereotactic radiosurgery (SRS) can be an alternative to surgery that can achieve high rates of local control with limited toxicity. From January 2005 to December 2013, 73 patients who developed pulmonary metastasis during period of adjuvant chemotherapy or follow-up were analyzed. 33 patients were treated by stereotactic radiosurgery using the body gamma-knife system. A total dose of 50 Gy was delivered at 5 Gy/fraction to the 50% isodose line covering the planning target volume, whereas a total dose of 70 Gy was delivered at 7 Gy/fraction to the gross target volume. The other 40 patients were treated by surgical resection. Four-year progression-free survival rate, four-year survival rate, median time of PRPFS (post-relapse progress-free survival) and PROS (post-relapse overall survival) in SRS group were parallel to that in surgical group. Patients tolerated gamma knife radiosurgery well. Our study demonstrates that SRS is well-tolerated with excellent local control and less complications. SRS should be considered as a potential option in patients with pulmonary metastases from osteosarcoma, especially in those who are medically inoperable, refuse surgery.

## Introduction

Osteosarcoma is the most common primary bone tumor in children and adolescents^1^. Approximately 15–20% of patients will have metastases at diagnosis^2,3^; 75% of these are in the lung^4,5^. Cure rates of nonmetastatic high-grade osteosarcomas have increased to 60–70% by addition of adjuvant and neoadjuvant multiagent chemotherapy to surgery^6^. Despite the development of effective treatment protocols, 30–40% of cases still relapse (metachronous metastases) and more than 80% of relapses are in the lungs^7,8^. Long-term post-relapse survival rate is under 20%^6–8^. For second-line chemotherapy, approximately 30 years have passed since four key anticancer drugs (methotrexate, adriamycin and cisplatin with or without ifosfamide) were introduced, development of new therapeutic drugs for osteosarcoma has stagnated and there is no standard for second-line therapy treating these patients who relapse^6,9–12^. A complete surgical removal of all pulmonary metastatic foci has been proven to be a best predictor of survival in patients with lung metastases from osteosarcoma in several studies^4,9,10,13–18^. Unfortunately, a considerable number of the patients cannot undergo surgery because of advanced disease or other medical conditions. Unfortunately, some of patients with pulmonary metastases from osteosarcoma will relapse with new sites of pulmonary disease despite metastatectomy. Many undergo multiple surgical resections for recurrent PM with the attendant morbidity and mortality associated with these invasive procedures. For patients with pulmonary oligometastases from sarcoma who are medically inoperable or who have had multiple prior resections, there is no accepted alternative to metastatectomy. Thus, development of novel techniques for osteosarcoma patients with pulmonary metastasis is highly desired.

Radiation therapy to the lung has been traditionally reserved to patients unfit for surgery. However, with the recent technological improvements, the indications for radiation therapy are broadening. Stereotactic radiosurgery (SRS) is based on the ability to use multiple radiation beams that intersect in a specific volume in three-dimensional space. One of the greatest differences between SRS and conventional radiotherapy is that in the former the software system allows for a precise delimitation of the volume to receive the highest radiation dose, which allows for steep fall-off dose gradients to adjacent tissue^19^. With satisfying local disease control rate and limited toxicity to surrounding tissues, it has been widely accepted in intracranial and noncranial neoplasms^20–27^, including primary or secondary pulmonary lesions^20,25,26,28–33^. Somehow, there are few data about the role of stereotactic radiosurgery in pulmonary metastases from osteosarcoma.

The stereotactic gamma-ray body therapeutic system (OUR™ Company, Shenzhen, China), or body gamma knife, is composed of radiation source, collimator and treatment couch as a complete package. This device has Co^60^ sources which can be utilized to form the sharp dose gradient and focused radiation to achieve target coverage while maintaining normal tissue tolerances in a rotating mechanism. Previous literatures have demonstrated inspiring outcome of patients suffered from lung and renal cancer treated with this advanced treatment protocol and device^34–37^. A retrospective study with a 2-years follow-up in our institute showed that in osteosarcoma patients with pulmonary metastases, stereotactic radiosurgery was effective and safe compared with surgical resection^38^. Thus, we reviewed the data from a 4-years follow-up to further evaluate role of stereotactic radiosurgery in treating pulmonary metastasis from osteosarcoma.

## Materials and Methods

### Eligibility criteria

From January 2005 to December 2013, patients with non-metastatic osteosarcoma of the extremity were diagnosed and treated at our institution with multi-agent chemotherapy reported in previous papers^39^. Patients relapsed with only pulmonary metastasis during period of postoperative chemotherapy or follow-up were eligible for this study in order to evaluate efficiency of stereotactic radiosurgery on pulmonary metastasis more precisely. All patients were treated at our institution. In chest CT scan, the number of pulmonary metastasis is defined from consensus among at least 3 radiological oncologists.

### Treatment for pulmonary metastasis

Surgical resection and stereotactic radiosurgery were administrated as previous reported in our literature^39^. Generally speaking, for metastasectomy, patients underwent a unilateral or bilateral thoracotomy at the same time. If found to be necessary during surgery, wedge resections were performed manually using vascular clamps and resorbable sutures. A lobectomy or a pneumonectomy was performed when necessary depending on the extension, number and site of the pulmonary lesions. Dissection of hilar lymph nodes was performed when necessary. After resection, all the specimens were checked by histological examination.

For stereotactic radiosurgery, the body gamma knife consists of a radiation source, collimator, and treatment bed. The head of radiation source is an iron ball rind with 30 Co^60^ sources scattered throughout the cavity of the primary collimator. The source body rotates horizontally around the central axis with the 30 bundles of gamma ray directed toward a focal target. In this study, three groups of chamber with collimator designs with aperture diameters of 3 mm, 12 mm, and 18 mm, respectively, were used; the full width at half-height of the dose-field range at the target was 10 mm, 30 mm, and 50 mm, respectively. As the aperture diameter of the collimator decreased, the density of the distributed dose increased, and the periphery dose decreased. Three groups of terminal collimators with different apertures direct the focusing of the radials. Tumors 1–10 cm in diameter could be treated using a combination of collimators with different aperture diameters. The focal dose rate at the initial source setting was 3 Gy/min. Finally, the treatment bed can move in X, Y, and Z directions and can automatically adjust the target to the focal point of the radials. All patients were immobilized using a stereotactic body frame with a vacuum pillow to create reproducible immobilization. Every patient underwent CT simulation ranging from the neck midline to 3 cm under the diaphragm with a CT-slide thickness of 5 mm and CT-slide interval of 5 mm. Selected patients with significant tumor motion (1 cm) were evaluated fluoroscopically; additional margins for tumor motion were added based on the results of the fluoroscopic analysis. Scanning images were sent directly to the planning system through the network with a 5 second scanning speed for each level. Gross target volume (GTV) was the primary tumor, clinical target volume (CTV) was allowed a 5 mm margin around the GTV; planning target volume (PTV) was created using pulmonary window, which allowed a 5 mm margin around the CTV. Low-speed CT was used, and did not consider the impact of respiratory move on inside target volume (ITV). The total radiation dose of 50%, 60%, and 70% isodose line were prescribed in 50, 60, and 70 Grey (Gy) correspondingly, covering 100% of the planning target volume (PTV), 90% of the clinical target volume (CTV), and 80% of the gross target volume (GTV) in 10 fractions. Radiotherapy was delivered 5 days per week for 2 weeks. The dose delivered to critical structures^34^ such as the main bronchi, esophagus, trachea, heart, and major blood vessels was required to be below 50 Gy (5 Gy/fraction), and the dose delivered to the spinal cord was required to be below 30 Gy (3 Gy/fraction). The patients’ posture were validated 2–3 times during the treatment, to ensure the positioning, planning, and treatment process accuracy.

### Treatment strategy for secondary relapse

For secondary relapse after SRS or surgical resection, therapy was not standardized. Surgical removal or SRS treatment of all detectable tumor foci was still recommended whenever feasible. If not feasible, administration of second-line chemotherapy or target therapy (sorafenib or everolimus) was conducted according to physician’s opinion.

### Data collection and assessments

Physical examination and routine laboratory were conducted every 2 weeks. Radiologic investigations were performed at every 2 month until progress (Assessed by RECIST 1.1).

The primary endpoint of the study was post-relapse progress-free survival (PRPFS), which was calculated from date of pulmonary metastasis until progress or last follow-up (Assessed by RECIST 1.1). The secondary endpoint was post-relapse overall survival (PROS), which was calculated from date of pulmonary metastasis until death or last follow-up. Toxicity was assessed according to the National Cancer Institute Common Toxicity Criteria (V3.0). The radiation reaction was classified as early or late side effects according to Radiation Therapy Oncology Group toxicity criteria.

For patients with secondary relapse, progress-free survival and overall survival after secondary relapse were also documented.

All patients provided written informed consent before study participation. The study was approved by the independent ethics committee, Sixth people’s Hospital, Shanghai JiaoTong University and conducted according to all applicable laws and regulations, good clinical practices and the ethical principles of the Declaration of Helsinki.

### Statistics

The evaluation of progress-free survival and overall survival after first/secondary relapse was performed using the Kaplan-Meyer method for calculating survival curves. Differences among the SRS and surgical-resection groups were compared by means of the ×2 test and t test. Multivariate analyses of survival including the variables that had correlated with PRPFS and PROS (ie, interval from to pulmonary metastasis, number of lesions, unilateral or bilateral lungs) were carried out using the Cox proportional hazards model. The significance was defined at a 2-sided, P value of <0.05. Statistical analysis was performed using the SPSS software, version 13.0 (SPSS Inc. Chicago, IL).

## Results

### Characteristics of patient suffered from pulmonary metastasis at relapse

Patient and tumor characteristics of all patients were summarized in Table 1. 73 patients were enrolled in this retrospective review with a 2.47 male-to female ratio and a mean age of 24 years (8 to 59 years). Limb salvage surgery was performed for 44 patients (60.2%) while amputation was performed for other 29 (39.8%). The most common primary bone sites were femur (40 patients, 54.8%), tibia (24 patients, 32.9%), humerus (7 patients, 9.6%) and fibula (2 patients, 2.7%).

**Table 1:** Characteristics of patients suffered from pulmonary metastasis.

Demographic Data	Resection	SRS	P value
**No of subjects**	40	33	
Gender			=0.43
Male	30	22	
Female	10	11	
**Age (years)**			
>18 years	28	18	=0.17
<18	12	15	
**Site**			
Femur	23	17	=0.634
Tibia	12	12	
Humerus	3	4	
Fibula	2	0	
**Surgery**			
Amputation	17	12	=0.59
Limb salvage	23	21	
**Histotype**			
Classic	36	30	=0.90
Others	4	3	
**Site of pulmonary lesions**			
Unilateral	24	13	=0.08
Bilateral	16	20	
**Num of pulmonary lesions**			
Solitary	18	10	=0.19
Multiple lesions (>2)	22	23	
**Time to relapse**			
≤2 years	28	28	=0.13
>2 years	12	5	

Median interval from initial treatment to pulmonary metastasis was 13 months (2 to 70.7 months). 34 patients (46.6%) developed lung metastases within 12 months after initial treatment, 22(30%) patients developed within 12 to 24 months and remaining 17(23.4%) patients developed later. 37 cases of pulmonary metastasis were unilateral involvement while the other 36 cases were bilateral. The median number of pulmonary metastatic foci was 2 (range from 1 to 9), The mean and median size of pulmonary metastatic foci were 10.6 mm and 9 mm range from 4 to 27 mm), respectively. 28 patients (38.3%) were solitary while 45 patients (61.7%) were involved multiple lesions.

### Treatment of Relapse

Surgery were reported in 40 patients, resulting in 17 wedge resections and 23 lobectomies. 33 patients underwent SRS treatment. For patients in surgical group, radiologically metastatic nodules were all proven to be metastasis from osteosarcoma histologically after resection. Table 1 demonstrated that age, gender, initial tumor site, histotype of tumor, method of surgery for initial tumor, time to relapse were well balanced between two groups. No difference was observed in aspects of pulmonary lesions’ site and pulmonary lesions’ number between two groups.

### Outcome in two groups

Until June 2017, the follow-up time after the relapse-treatment for surgical-group and SRS group was 5 to 127 months (median 36 months) and 7 to 86 months (median 18 months) respectively. In patients without progression or still alive after relapse-treatment, their minimum follow-up time had reached 4 years.

**Figure 1:**
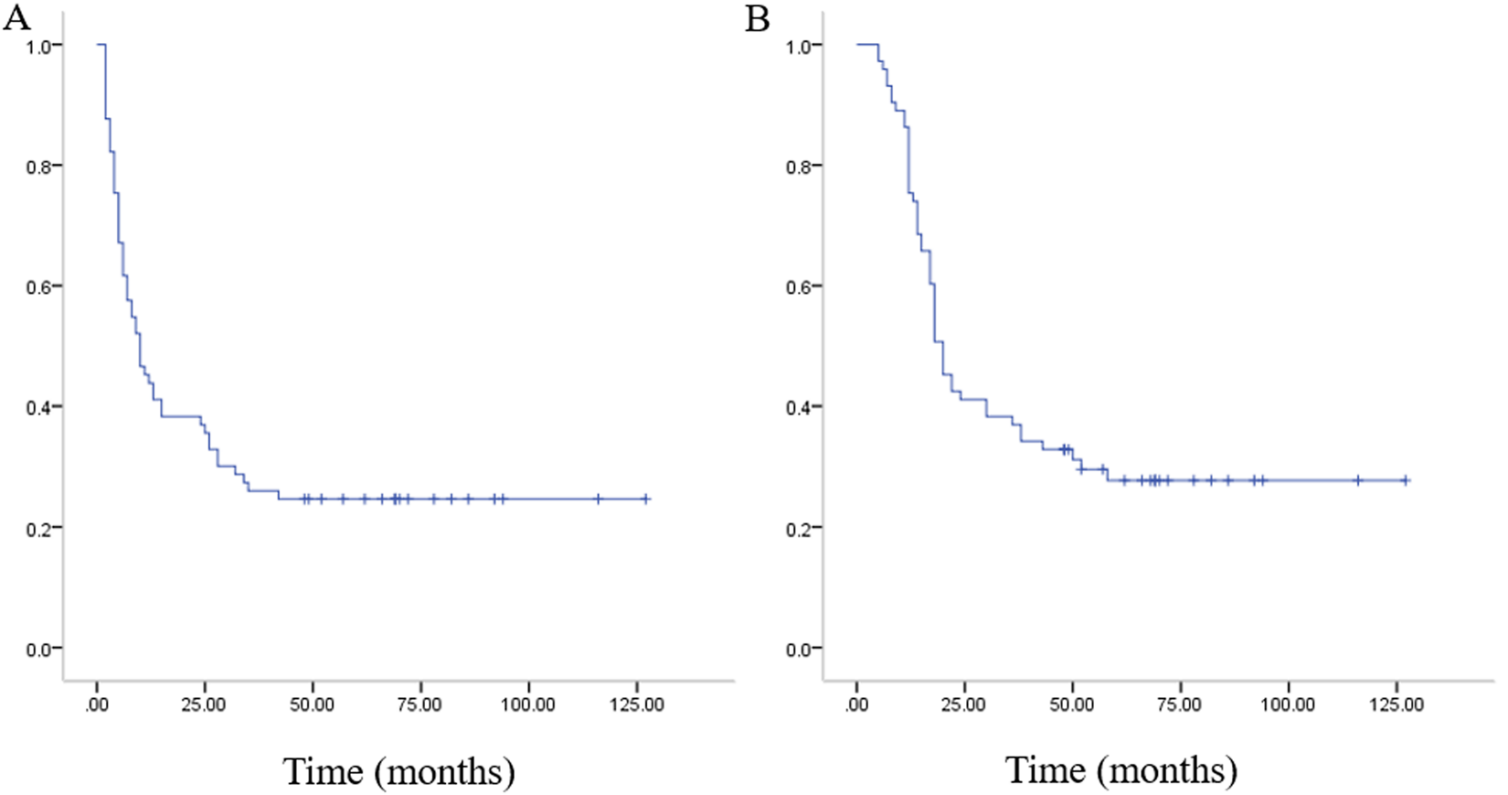
(**A**) Kaplan–Meier plots of post-relapse progress-free survival for all patients. (**B**) Kaplan–Meier plots of post-relapse overall survival for all patients.

Of 73 patients, the median time of PRPFS and PROS was 10 months (range from 2 to 127 months) and 20 months (range from 5 to 127 months), respectively (Fig. 1A and B).

**Figure 2:**
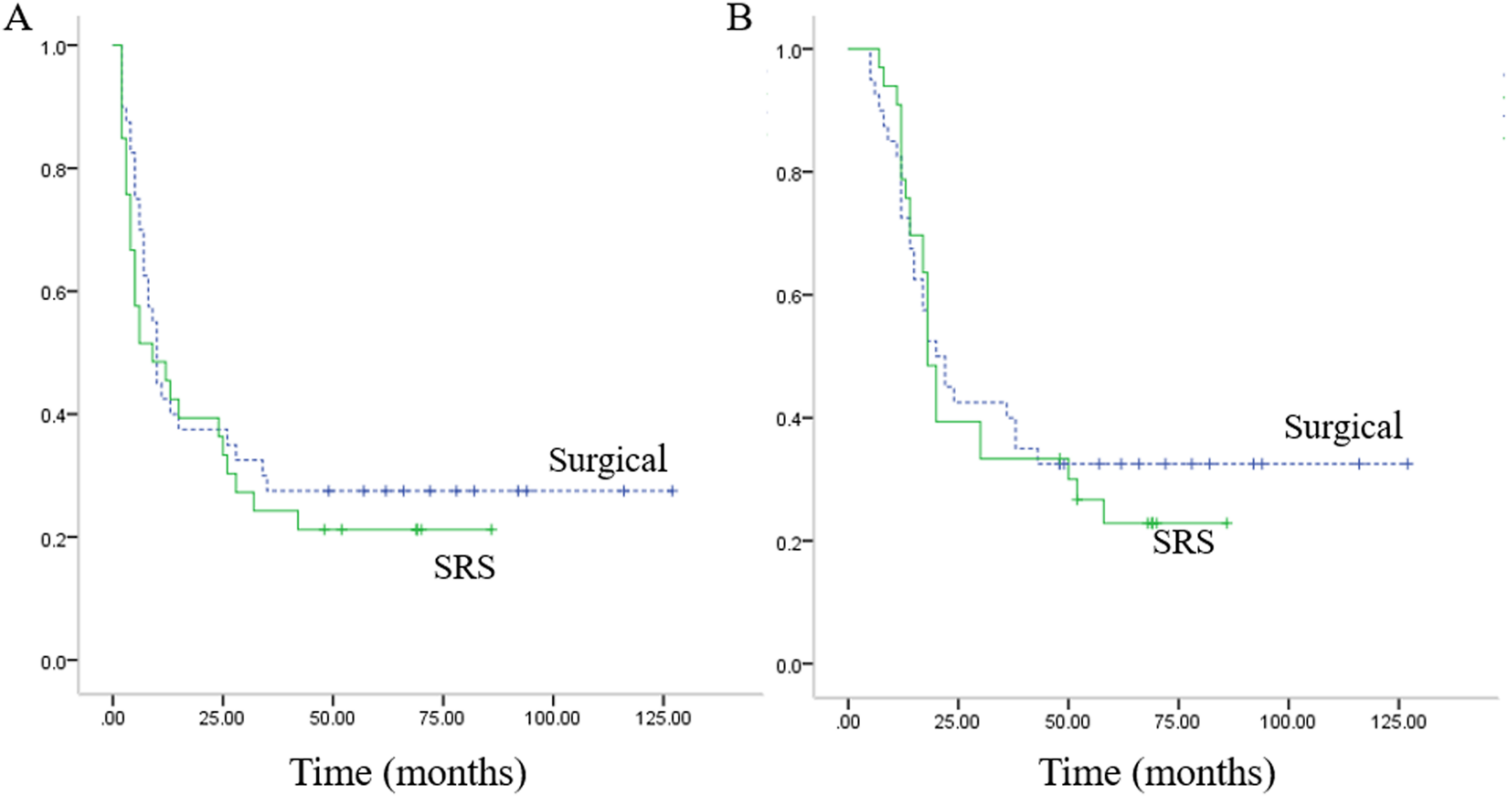
(**A**) Kaplan–Meier plots of post-relapse progress-free survival for surgical-group and SRS-group. (**B**) Kaplan–Meier plots of post-relapse overall survival for surgical-group and SRS-group. Post-relapse progress-free survival and post-relapse overall survival in SRS group were parallel to that in surgical group (P > 0.05).

The four-year progression-free survival rate was 27.5% (11/40) in surgical group and 21.2%(7/33) in SRS group. The four-year survival rate was 32.5% (13/40) in surgical group and 33.3%(11/33) in SRS group. The median time of PRPFS in surgical-group and SRS group was 10 months and 9 months respectively. The median time of PROS in surgical-group and SRS group was 20 months and 18 months respectively (Fig. 2A and B). Differences in median time of PRPFS, median time of PROS, four-year progression-free survival rate and four-year survival rate between two groups were not significant (P > 0.05).

Cox proportional hazards model indicated that time to relapse and number of pulmonary lesions were of prognostic value in PRPFS and PROS. And these two factors were well balanced between two groups as mentioned in Table 1.

### Pattern, treatment and outcome of secondary relapse

Until June 2017, follow-up information demonstrated fifty-five secondary relapse. At recurrence, lungs were again involved in thirty-eight cases (nineteen in surgical and nineteen in SRS). Five pulmonary metastasis were combined with bone metastasis (four in surgical and one in SRS) and two were combined with soft tissue metastasis (two in SRS). Proportion of cases with relapse outside of lungs increased, with seven cases involved bone (three in surgical and four in SRS) and three local recurrence at initial tumor site (two in surgical and one in SRS). For twenty-two pulmonary relapse in SRS group, four were within the filed of GTV, two were within the field between GTV and CTV while the rest sixteen were out the field of SRS treatment.

**Figure 3:**
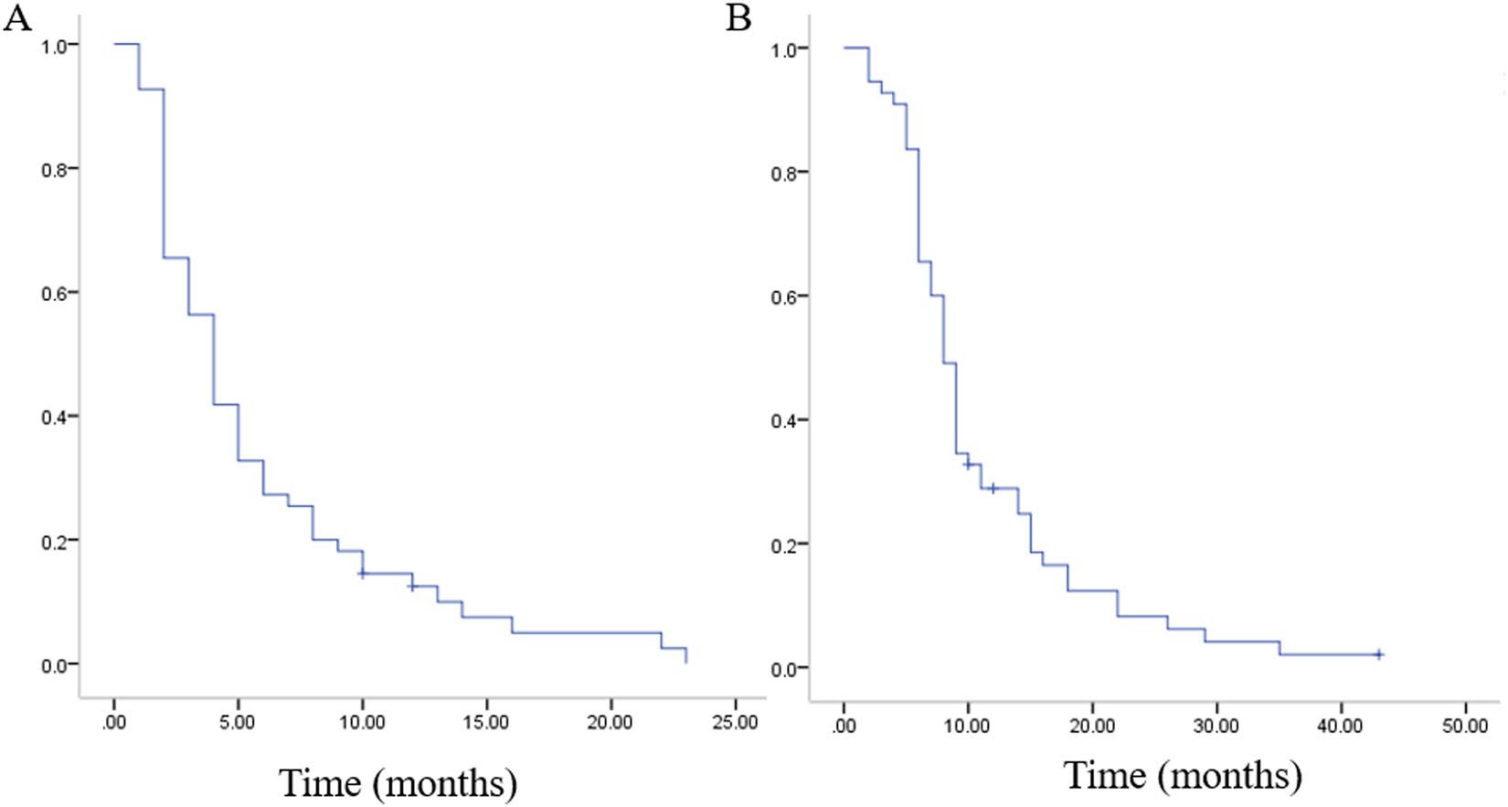
(**A**) Kaplan–Meier plots of progress-free survival for patients after secondary relapse. (**B**) Kaplan–Meier plots of overall survival for patients after secondary relapse.

**Figure 4:**
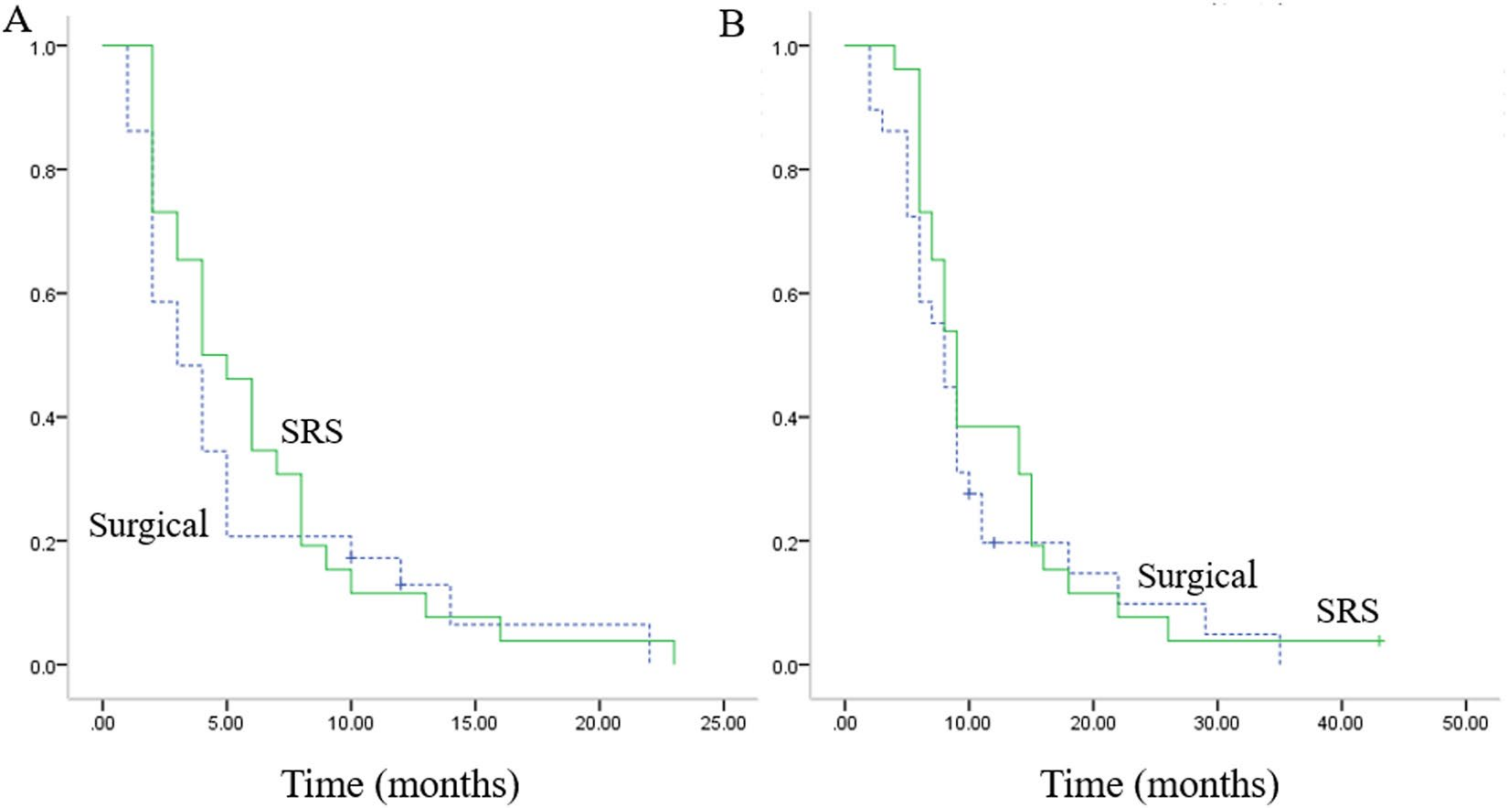
(**A**) Kaplan–Meier plots of progress-free survival for patients with secondary relapse. (**B**) Kaplan–Meier plots of overall survival for patients with secondary relapse. After SRS treatment, outcome of patients with secondary relapse was parallel to that after surgical treatment (P > 0.05).

Second-line chemotherapy or target therapy were reported for 35 of 55 secondary relapses. SRS/metastasectomy for secondary pulmonary relapse were performed in 12 patients. Conventional radiation therapy were administrated in 5 cases of bone metastasis. All three cases of local recurrence chose amputation. Survival of patients who had a second relapse were: 4 months for progress-free survival time (3 months in surgical group, 4 months in SRS group) and 8 months for over all survival time (8 months in surgical group, 9 months in SRS group), which indicated a poorer prognosis (Figs 3A and B and 4A and B). Treatment option for first relapse (SRS or metastasectomy) did not affect outcome after second relapse (Fig. 4A and B, P > 0.05). Besides, it is noteworthy that among seven patients who had a second remission more than 12 months, four chose SRS/metastasectomy treatment (2 chose metastasectomy, 2 chose SRS) after secondary relapse.

### Complications for SRS and surgical resection

**Figure 5:**
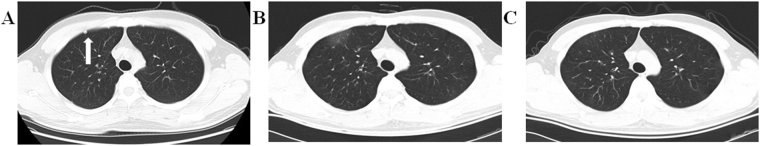
Radiation pneumonitis occur 2 months after SRS treatment. (**A**) Pulmonary metastasis before SRS treatment (Pointed by white arrow). (**B**) One month after SRS treatment, CT scan showed radiation pneumonitis without any symptom from the patient. (**C**) Without medical intervention, radiation pneumonitis resolved two months later.

No acute or late severe pulmonary toxicity (Grade 3 to 5) occurred in SRS group. For 39 cases of SRS treatment (33 for first relapse and 6 for secondary relapse), grades 1 to 2 radiation pneumonitis were recorded in 14 cases (10 patients with grade 1 while 4 patients with grade 2), account for 35.8%. All radiation pneumonitis vanished within 3 months without any treatment (Fig. 5). Other complications such as development of pleural effusions, hemoptysis, tracheoesophageal fistula, pericardial effusions, and pneumothoraces were not observed.

**Figure 6:**
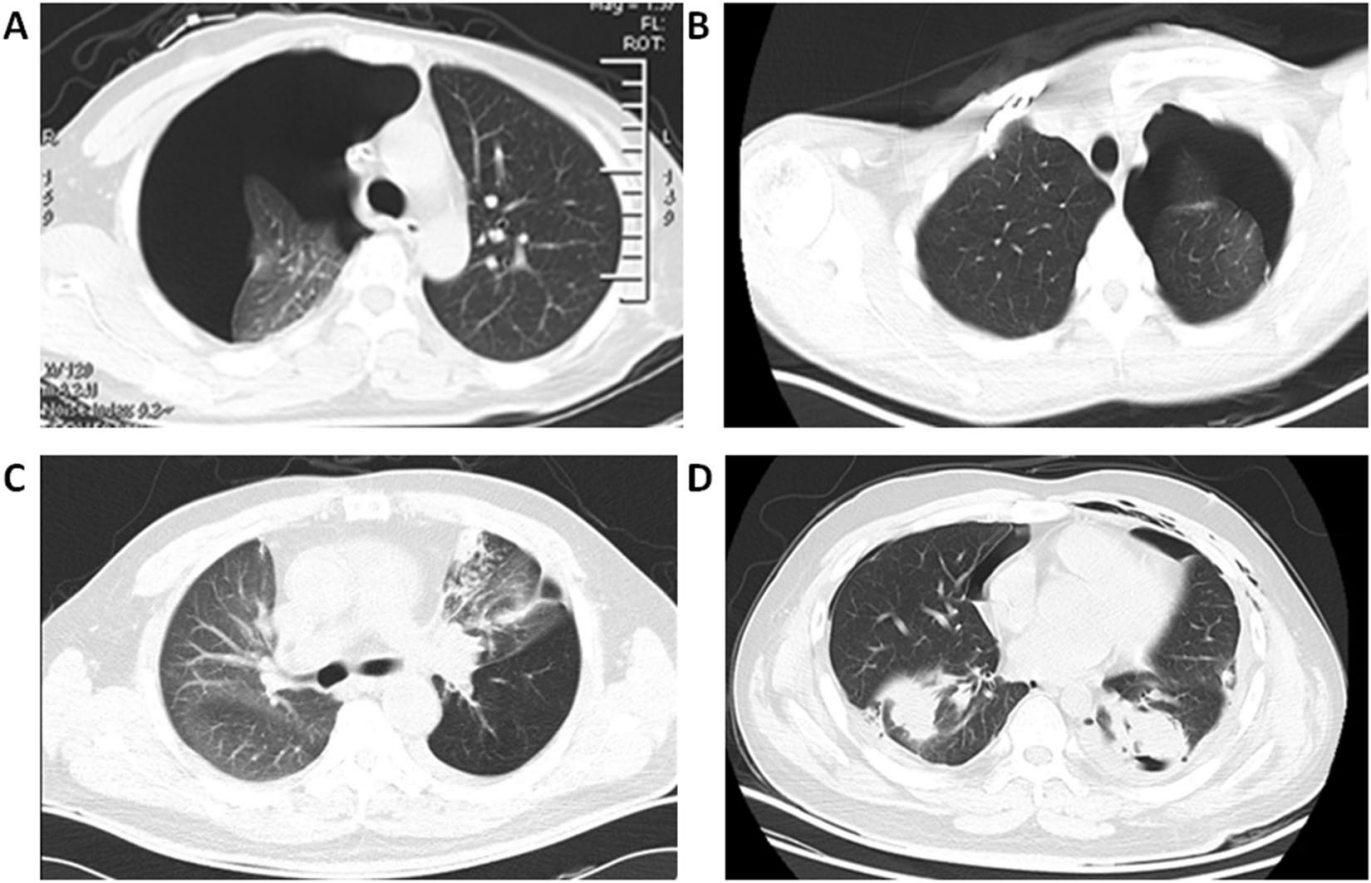
Complications after surgical resection. (**A**) Persistent pneumothorax (nearly 90%). (**B**) Persistent pneumothorax (<30%). (**C**) Pneumonia. (**D**) Intrapulmonary hematomas.

**Figure 7:**
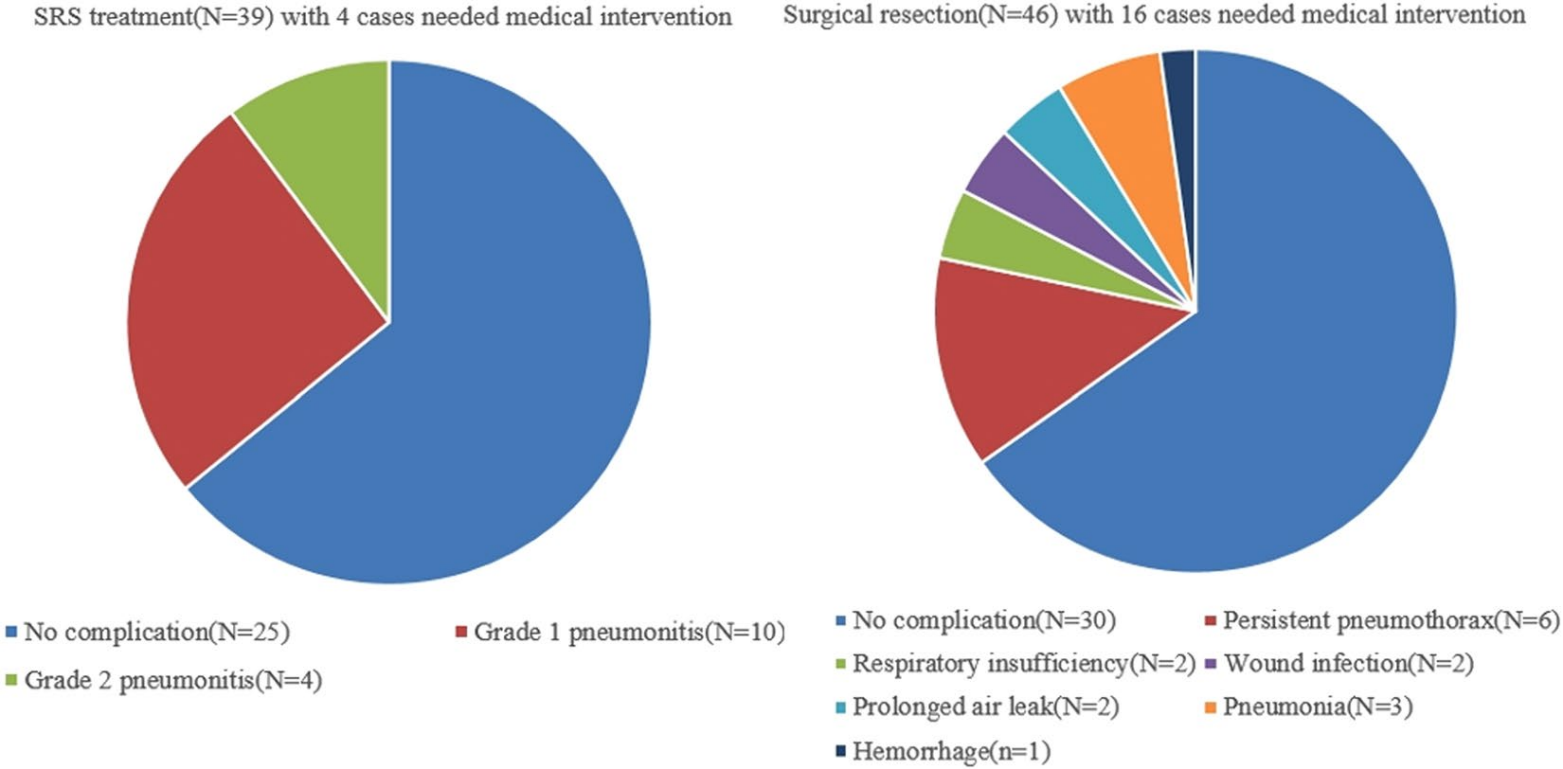
Surgical group had more cases with complications in needed of medical intervention (16 out of 46) than SRS group (4 out of 39) (P = 0.02).

For 46 cases of surgical resection (40 for first relapse and 6 for secondary relapse), no surgery-related deaths were recorded. There were 6 cases of persistent pneumothorax, which was resolved by keeping the drainage *in situ* for a longer period of time (10–15 days) and by placing a unidirectional valve removed once the pneumothorax resolved. Other complications were two cases of respiratory insufficiency, two cases of superficial wound infection, two cases of prolonged air leak, three cases of pneumonia and one case of hemorrhage (Fig. 6). All the 16 cases recovered within 2 months under aggressive medical intervention. There were more cases with complications in need of medical intervention (16 out of 46) in surgical group than that (4 out of 39) in SRS group (P = 0.02, Fig. 7).

## Discussion

Osteosarcoma frequently metastasize to the lungs and can lead to high rates of morbidity and mortality. Chemotherapy in these patients is often of limited benefit. For pulmonary lesions who are medically operable, metastatectomy is a well-established standard treatment that is associated with high rates of local control. Surgical resection, however, can be associated with significant morbidity, and some patients still develop subsequent lung metastases even after a complete resection of their initial metastases. There is no standardized approach for treating patients with pulmonary metastases from osteosarcoma who are medically inoperable, refuse surgery, or who have already undergone multiple prior metastatectomies. Metastatic characteristics of osteosarcoma are demonstrated as affinity for the lung, usually small volume and often located at peripheral parts of the lung. SRS-induced lung injury, such as radiation pneumonitis, is correlated with tumor size and location^20^. Tumors greater than 50 ml in size and those associated with the central airways are with higher possibility of radiation pneumonitis^29,30^. Thus, stereotactic radiosurgery is one promising potential alternative to resection. Somehow, limited data currently exist assessing the efficacy and safety of SRS for treating osteosarcoma pulmonary metastases. No prospective clinical trials have been published on SRS for osteosarcoma metastasis to the lungs.

We previously confirmed that stereotactic radiosurgery treatment by body gamma knife on osteosarcoma patients with pulmonary metastasis could yield an outcome which was equivalent to that from surgical resection, accompanied with less complication on a 2-year follow-up^38^. From a 4-year follow-up, we demonstrated that in treating pulmonary metastasis from osteosarcoma, SRS has excellent control compared with surgical resection. Although there was an one-month difference in PRPRS and a two-months difference in PROS with better results for surgical-group, the difference is not significant and might be contributed to a higher proportion of patients with one pulmonary metastatic lesion in surgical group (18 out of 40, while 10 out of 33 in SRS group), as Cox proportional hazards model indicated that number of pulmonary lesions was a key factor which could affect PRPFS and PROS. Further more, treatment pattern for first relapse (SRS or surgical resection) did not affect the survival of patients with secondary relapse.

In our series, toxicity of body gamma-knife radiosurgery was tolerable, with very few grade 2 acute or late toxicities, and no grade ≥3 toxicity or treatment-related death. The proportion of complication in need of medical intervention was much less in SRS group compared with resection.

How to improve the outcome of secondary relapse still is a tough challenge. Data in our study demonstrated that second-line chemotherapy or target therapy were mainstream treatment after secondary relapse, with a much less obvious effect than that of first-line chemotherapy. It is noteworthy that among seven patients who had a second remission more than 12 months, four chose SRS/metastasectomy treatment after secondary relapse. Previous reports^9,10,14^ demonstrated repeat pulmonary resection for patients with recurrent metastasis from osteosarcoma would improve their survival. Therefore, in case of secondary pulmonary relapse, choice of resection or SRS should be recommended whenever feasible.

There are several limitations to this study. We can not exclude patient selection bias on the retrospective nature of our analysis. Besides, patients in China afford the fee of SRS treatment by themselves, not by the medical insurance. This might make some patients eligible for SRS choose surgery. Furthermore, the relatively modest patient numbers (only 73) may limit the generalizability of our findings. Additionally, since our study was the first time to investigate efficacy of body gamma knife in treatment of pulmonary metastasis from osteosarcoma, we took the prescribed radiation dosage reported in Xia’s study, which investigated NSCLC, as a reference. The radiation dosage might not be optimal. Moreover, our study used slow CT simulation and fluoroscopy to compensate for tumor motion during breathing without 4D CT or ITV planning. Although among 11 patients in SRS group with significant tumor motion (>1 cm)during breathing, seven patients had secondary relapse as pulmonary recurrences and they were well balanced (P = 0.88) in patients with significant tumor motion (3 out of 11) or not (4 out of 22), the small number (N = 7) of secondary pulmonary recurrences after SRS treatment might limit statistical power to show the difference. We still believe better evaluation and targeting of tumor motion based on 4D CT or ITV planning could further reduce the risk of local recurrence.

Despite those shortcomings mentioned, based on a 4-year follow-up, our study demonstrated that stereotactic radiosurgery (SRS) is a potentially alternative treatment for pulmonary metastasis of osteosarcoma after failure of adjuvant chemotherapy, especially for those patients who were medically unfit for a resection, or who refused surgery. Further studies to confirm the results, especially prospective clinical trials, focusing on the efficiency of SRS treatments for osteosarcoma patients with pulmonary metastasis compared with surgical resection, should be strongly considered.
